# Hilar and Mediastinal Lymphadenopathy as a Potential Manifestation of Azathioprine-Associated Hypersensitivity Syndrome

**DOI:** 10.3390/ph19070997

**Published:** 2026-06-27

**Authors:** Mairi Ziaka

**Affiliations:** 1 Department of Emergency Medicine, Inselspital, University Hospital, University of Bern, 3010 Bern, Switzerland; mairi.ziaka@gmail.com; 2 Department of Internal Medicine, Thusis Hospital, 7430 Thusis, Switzerland

**Keywords:** azathioprine, azathioprine-associated hypersensitivity syndrome, autoimmune hepatitis, DRESS, hypersensitivity reaction, lymphadenopathy, skin manifestations

## Abstract

Azathioprine-associated hypersensitivity syndrome is a rare, idiosyncratic, dose-independent reaction that typically develops within the first four weeks of therapy. The clinical presentation includes a broad spectrum of symptoms such as fever, gastrointestinal manifestations including abdominal pain, nausea, and vomiting, cutaneous involvement, arthralgias, myalgias, and organ dysfunction, particularly affecting the liver and kidneys. Laboratory findings typically include leukocytosis, neutrophilia, and elevated C-reactive protein (CRP), whereas eosinophilia is usually absent. To date, lymphadenopathy has not been described as a manifestation of this syndrome. In this report, we describe the case of a 75-year-old female patient who developed a probable azathioprine-associated hypersensitivity syndrome three weeks after treatment initiation and who, in addition to systemic manifestations of the syndrome, presented with bihilar and mediastinal lymphadenopathy, which may represent a previously unreported feature of this condition, and we discuss potential differential diagnoses.

## 1. Introduction

Azathioprine belongs to the thiopurine drugs and is an immunosuppressive purine nucleoside analogue widely used in the treatment of hematological, autoimmune, and inflammatory diseases, including autoimmune hepatitis (AIH), rheumatoid arthritis, systemic lupus erythematosus, Crohn’s disease, inflammatory bowel disease, multiple sclerosis, and antineutrophil cytoplasmic antibody (ANCA)-associated vasculitis, where it is commonly employed as a first-line therapy in combination with corticosteroids [1,2,3,4]. Azathioprine is an inactive prodrug that is rapidly converted extracellularly to 6-mercaptopurine (6-MP) by glutathione S-transferase. 6-MP is subsequently metabolized through different pathways: one fraction is converted to thiouric acid (6-TUA) by xanthine oxidase (XO), whereas another fraction undergoes intracellular metabolism to active metabolites, including 6-thioguanine nucleotides (6-TGNs), through the actions of hypoxanthine phosphoribosyltransferase (HPRT) and thiopurine methyltransferase (TPMT) [5]. The immunosuppressive and immunomodulatory properties of azathioprine are mediated by intracellular inhibition of purine synthesis, disruption of deoxyribonucleic acid (DNA) and ribonucleic acid (RNA) production, and impairment of highly proliferative cells such as T and B lymphocytes, as well as a reduction in circulating B and T lymphocyte populations, and pronounced CD56dimCD16+ natural killer cell depletion with concomitant interferon (IFN)-γ deficiency [6,7,8].

Adverse effects such as hepatotoxicity, gastrointestinal intolerance, pancreatitis, and myelosuppression are well recognized and occur in approximately 10–15% of patients receiving azathioprine [1,2,5,9]. In addition, due to its immunosuppressive and genotoxic/mutagenic properties, azathioprine is associated with an increased risk of malignancies, including lymphoproliferative and myeloproliferative cancers, urinary tract cancers, skin malignancies, and cervical cancer; therefore, the indication for azathioprine should be carefully individualized [10,11,12,13].

Previous research highlights that genetic polymorphisms in the TPMT gene contribute significantly to the pathophysiology of azathioprine-induced adverse reactions. Heterozygous individuals carrying a mutant TPMT allele exhibit intermediate TPMT activity, whereas homozygous or compound heterozygous individuals show markedly reduced or absent TPMT activity, leading to accumulation of 6-TGNs and an increased risk of adverse effects [2,14,15,16,17]. True hypersensitivity reactions secondary to azathioprine administration represent a rare complication, observed in about 2% of treated patients. These reactions occur in an idiosyncratic, dose-independent manner and typically develop within the first four weeks of therapy [1,9]. The pathophysiology of azathioprine hypersensitivity syndrome is complex and not fully understood. However, it appears to develop independently of TPMT activity, represents a delayed hypersensitivity response, and likely involves type III and type IV hypersensitivity reactions as well as neutrophil activation [18,19,20,21,22]. The clinical presentation of the syndrome includes systemic symptoms such as fever, nausea, abdominal discomfort, arthralgias, cutaneous manifestations, and laboratory abnormalities, which may be misinterpreted as a relapse of the underlying autoimmune disease or as a systemic infection [1,20]. Nevertheless, it should not be overlooked that azathioprine hypersensitivity reactions can be life-threatening, with progression to anaphylactic shock, likely associated with type I IgE-mediated anaphylaxis or non-IgE-mediated anaphylactoid reactions [18,20,21].

Drug reaction with eosinophilia and systemic symptoms (DRESS) is a serious, delayed drug-induced hypersensitivity reaction that is mediated by T cells and occurs in an idiosyncratic manner. It typically presents with fever, cutaneous manifestations, and facial edema, along with lymph node enlargement and signs of internal organ involvement. Laboratory findings often include eosinophilia and atypical lymphocytes. Although it can affect multiple organs, the liver, kidneys, lungs, pancreas, and heart are most frequently involved [23].

Here, we report the case of a 75-year-old woman who developed fever, nausea, vomiting, arthralgia, and a maculopapular, scaly rash three weeks after initiation of azathioprine for the treatment of AIH. Thoracic imaging revealed bihilar and mediastinal lymphadenopathy, which may represent a previously underreported manifestation of azathioprine-associated hypersensitivity. Alternatively, this finding broadens the differential diagnosis to include DRESS syndrome.

## 2. Case Presentation

The patient was admitted on an emergency basis following referral by her general practitioner due to recurrent vomiting for four days. She reported vomiting occurring immediately after intake of medications or meals. She denied nausea or additional abdominal complaints. There were no changes in bowel habits, although stool frequency was reduced due to decreased oral intake.

At presentation, the patient additionally complained of acute lumbar back pain, which appeared to worsen when sitting up. She also experienced new-onset bilateral heel pain while standing, as well as bilateral knee pain.

The patient’s past medical history was notable for AIH and type 2 diabetes mellitus. Initial therapy for AIH consisted of budesonide 9 mg daily, which led to a significant improvement in liver enzymes. Consequently, corticosteroid therapy was tapered, and at the time of admission to our hospital, the patient was receiving prednisolone 10 mg daily and azathioprine 100 mg daily, which had been initiated three weeks prior to the current presentation as steroid-sparing maintenance therapy for AIH. Moreover, approximately 3 weeks prior to presentation, the patient received antibiotic therapy with fosfomycin, nitrofurantoin, and ciprofloxacin for a urinary tract infection.

### 2.1. Assessment, Treatment, and Clinical Course

On admission, the patient was cardiopulmonary stable, presenting with a slightly reduced general condition. The predominant clinical findings were acute lumbar back pain and arthralgias involving both knees and heels.

Laboratory evaluation revealed elevated transaminases and cholestatic parameters, impaired renal function, along with a markedly elevated CRP and lipase, while the leukocyte count was slightly elevated (Table 1). Additionally, hyponatremia and hypocalcemia were present.

A computed tomography (CT) scan of the thorax and abdomen showed no evidence of an infectious focus or biliary obstruction, but revealed multiple prominent and partially enlarged mediastinal and bilateral hilar lymph nodes, including a precarinal lymph node with a short-axis diameter of up to 11 mm. In addition, multiple bilateral peribronchovascular pulmonary nodules measuring up to 5 mm were identified.

Serologies for Epstein–Barr virus (EBV), cytomegalovirus (CMV), and parvovirus B19 were negative for acute infection. Blood cultures remained negative, and polymerase chain reaction (PCR) testing for respiratory viruses was also negative. Angiotensin-converting enzyme (ACE) levels were within the normal range.

**Figure 1:**
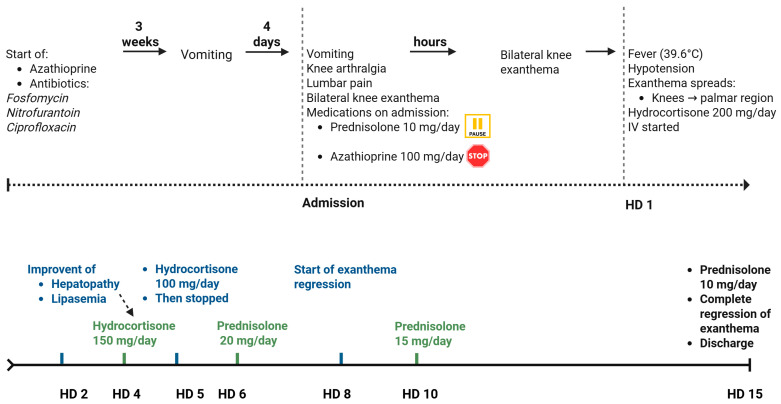
Timeline of clinical course following azathioprine initiation.

Given the elevated lipase levels and after consultation with the gastroenterology service, azathioprine was discontinued. On hospital day 1, the patient developed a fever up to 39.6 °C and hypotension with a nadir of 83/57 mmHg. Intravenous hydrocortisone (50 mg four times daily) was initiated, leading to a rapid improvement in inflammatory markers and cholestatic parameters (Table 1), with stabilization of hemodynamics and resolution of fever, allowing transition to oral prednisone 20 mg daily (Figure 1).

**Figure 2:**
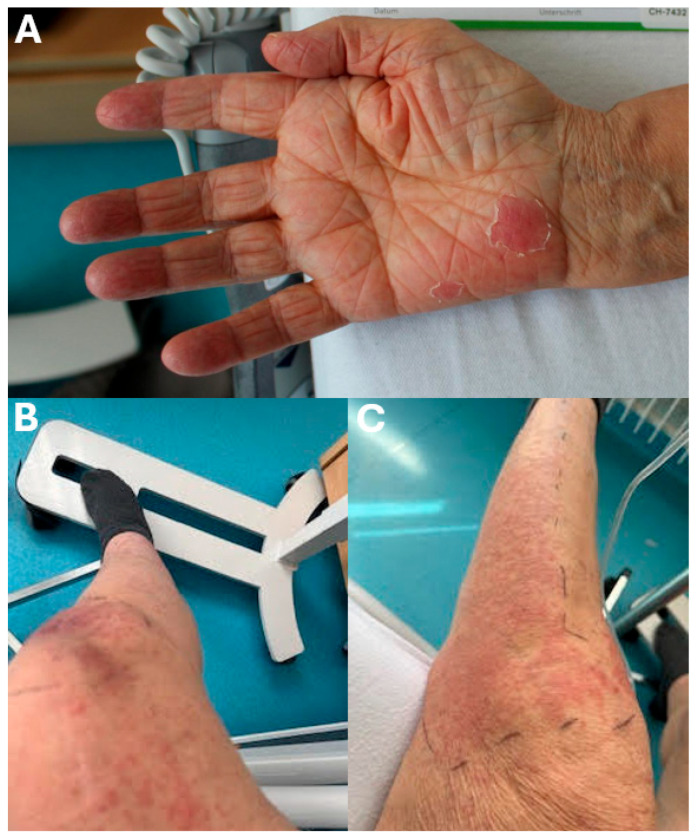
(**A**) Erythematous, desquamating plaques on the palmar aspect of the hand with superficial epidermal detachment. (**B**,**C**) Confluent erythematous maculopapular exanthem involving both lower extremities.

During the hospital course, the patient developed a painless, non-pruritic maculopapular rash involving both lower legs and the palms (Figure 1 and Figure 2), which resolved under ongoing steroid therapy.

The inpatient course was complicated by significant glycemic variability. Despite corticosteroid therapy, the patient experienced recurrent nocturnal hypoglycemia, necessitating a reduction in basal insulin dosage. Following insulin adjustment and gradual tapering of corticosteroids, blood glucose levels stabilized throughout the day.

### 2.2. Diagnostic Considerations

Overall, the clinical presentation, characterized by emesis, exanthema, arthralgias, predominantly cholestatic hepatopathy, elevated lipase levels, and acute kidney injury, was primarily interpreted as a hypersensitivity reaction to azathioprine. Notably, the presence of bihilar and mediastinal lymphadenopathy cannot be readily explained by azathioprine hypersensitivity alone.

A DRESS syndrome was considered in the differential diagnosis, with a RegiSCAR (Severe Cutaneous Adverse Reaction scoring) score of 5 points (Table 2). However, eosinophilia was never documented, which may have been masked by the patient’s ongoing long-term corticosteroid therapy. Measurement of eosinophilic cationic protein was also normal.

### 2.3. Outcome and Follow-Up

At the time of discharge, the patient was receiving prednisolone 10 mg daily (Figure 1). Following consultation with the gastroenterology team, mycophenolate mofetil therapy was initiated. Laboratory follow-up showed no signs of systemic inflammation, with only mildly elevated γ-glutamyl transferase (gamma-GT) and lipase levels.

## 3. Discussion

Azathioprine is a pharmacologically inactive prodrug that is initially metabolized to 6-MP, which is subsequently converted into active metabolites, including 6-TGNs and methylnitroimidazole. While 6-TGNs mediate the therapeutic effects of azathioprine, they are also implicated in the pathophysiology of dose-dependent adverse effects. In parallel, 6-MP undergoes further metabolism to inactive metabolites through the enzymatic activity of TPMT and XO. Reduced TPMT activity, resulting from single-nucleotide polymorphisms on chromosome 6, or inhibition of XO, increases the risk of adverse events and drug toxicity. Nevertheless, hypersensitivity reactions are thought to be mediated primarily by the imidazole side chain of azathioprine rather than by TPMT-dependent pathways, as evidenced by the absence of such reactions with 6-MP, which lacks the imidazole moiety [24,25]. Nonetheless, the pathophysiology of azathioprine hypersensitivity syndrome remains incompletely understood; it is hypothesized to involve immune-complex-mediated (type III) and T-cell-mediated (type IV) hypersensitivity reactions, with neutrophils playing a fundamental role in the pathogenesis of the syndrome [1].

In patients with AIH, clinical guidelines recommend combined immunosuppressive therapy with a glucocorticoid and azathioprine as first-line treatment, with the primary goal of achieving complete biochemical remission, defined by normalization of aminotransferases and immunoglobulin G (IgG) levels, which is attained in approximately 38.8–70.5% of patients after six months of therapy. However, adverse effects such as hematologic abnormalities, gastrointestinal complications, and arthralgias lead to treatment discontinuation in about 20% of patients [4]. Hypersensitivity reactions occur early after initiation of azathioprine therapy, typically within days to four weeks [9,24]. Patients with azathioprine hypersensitivity syndrome exhibit a variety of symptoms, including fever, chills, gastrointestinal discomfort with abdominal pain, nausea, and vomiting, arthralgias, myalgias, cutaneous manifestations, acute kidney injury, hepatic involvement, and, rarely, circulatory compromise. Common laboratory findings include leukocytosis, neutrophilia, and elevated CRP. Interestingly, in a study including 290 patients receiving azathioprine for ANCA-associated vasculitis, 25 developed azathioprine-associated hypersensitivity syndrome, and none presented with eosinophilia [1,20].

Our patient presented with fever, hypotension, arthralgias, vomiting, and a maculopapular rash involving the lower extremities and palmar surfaces of the hands, along with elevated transaminases and cholestatic parameters, significant lipasemia, worsening renal function, leukocytosis with neutrophilia, and normal eosinophils. However, CT of the thorax additionally revealed bihilar and mediastinal lymphadenopathy, which, to the best of our knowledge, is not a typical finding in azathioprine-associated hypersensitivity syndrome.

Mediastinal lymphadenopathy can occur in the setting of various benign disorders, including infectious and non-infectious granulomatous diseases such as tuberculosis, fungal infections, and sarcoidosis; chronic inflammatory conditions, including interstitial lung diseases; autoimmune diseases; and drug-induced hypersensitivity reactions. Malignant etiologies include lymphoproliferative disorders such as lymphoma, as well as lung cancer and extrapulmonary malignancies such as breast and esophageal cancer [23,26,27]. In our patient, despite the absence of hypercalcemia, and given that sarcoidosis is one of the most common causes of bilateral hilar lymphadenopathy and that bilateral hilar lymphadenopathy is highly suggestive of sarcoidosis [28], we performed ACE measurement, which was within the normal range, making sarcoidosis less likely. Moreover, in the absence of B symptoms (i.e., persistent fever, night sweats, and significant weight loss) and in the presence of marked hyperlipasemia and hepatitis, both lymphoma and tuberculosis were ranked lower in the differential diagnosis [29,30]. Furthermore, due to the increased risk of endogenous herpesvirus reactivation during ongoing azathioprine therapy [6], we tested EBV and CMV serologies, which were negative for acute infection. However, blood PCR was not performed, representing a major limitation of our diagnostic workup. Parvovirus B19 serology indicated a past infection. Finally, the presence of multiple small bilateral peribronchovascular pulmonary nodules was considered a nonspecific finding, as the vast majority of pulmonary nodules, approximately 95%, are benign, most commonly representing granulomas or intrapulmonary lymph nodes, particularly when the nodules are small in size [31].

Considering the onset of symptoms three weeks after initiation of azathioprine and antibiotic therapy, and the presence of fever, skin rash, multiorgan involvement, and lymphadenopathy, a drug hypersensitivity reaction resembling DRESS syndrome was considered as an additional differential diagnosis, although without eosinophilia or atypical lymphocytes. Based on our previous publication of a case of probable atypical DRESS syndrome [23], we performed measurement of eosinophilic cationic protein, which was also within normal ranges. When assessed using the RegiSCAR system, our patient achieved a total of five points, leading to classification as a probable case of DRESS despite the lack of peripheral eosinophilia. This diagnostic framework integrates a range of clinical features, including fever, lymph node enlargement, and the presence as well as extent of cutaneous involvement, with laboratory parameters such as eosinophil counts, presence of atypical lymphocytes, and evidence of internal organ involvement, and applies a weighted scoring approach to stratify cases as negative, possible, probable, or definite DRESS [23,32].

Despite the classification of our case as “probable” according to the RegiSCAR criteria, we considered DRESS syndrome less likely based on the following observations. First, there was an absence of eosinophilia, and levels of eosinophilic cationic protein were normal. Eosinophilia is one of the most common findings in DRESS syndrome, with a reported incidence ranging from 52% to 95%, and it forms part of the RegiSCAR criteria. It should be noted that eosinophilia may appear with a delay of 1–2 weeks after disease onset. However, our patient did not exhibit eosinophilia on repeated measurements over a two-week period (Table 1) [33,34]. Secondly, she did not exhibit peripheral lymphadenopathy. Enlarged lymph nodes are a common finding in patients with DRESS, with the most commonly affected sites being the cervical, inguinal, and axillary regions, whereas salivary gland involvement has also been reported. Mediastinal lymphadenopathy may occur in the context of DRESS syndrome, even as an isolated manifestation; however, it appears to be a rare and atypical feature [23,35,36]. Thirdly, DRESS syndrome is characterized by a prolonged clinical course ranging from 3 to 18 weeks, with a mean resolution time of approximately 7 weeks. Our patient showed rapid clinical and laboratory improvement after discontinuation of azathioprine and an increase in the dose of corticosteroid therapy [37].

Overall, the findings are most consistent with azathioprine-associated hypersensitivity syndrome, particularly in light of the resolution of clinical symptoms and the marked improvement in laboratory parameters following discontinuation of azathioprine and escalation of glucocorticoid therapy. Given the rarity of this syndrome and the extremely limited available data, lymphadenopathy may represent a rare and underreported manifestation. Alternatively, the observed lymphadenopathy may represent an incidental finding unrelated to azathioprine-associated hypersensitivity and could be attributable to a different underlying etiology. Although DRESS syndrome was considered less likely, the absence of eosinophilia and normal eosinophilic cationic protein levels could potentially be explained by chronic immunosuppression of eosinophils due to prolonged glucocorticoid therapy. Repeat CT of the thorax and lymphocyte proliferation testing after glucocorticoid discontinuation are planned and may help further clarify this complex clinical presentation.

Our diagnostic approach has several limitations. First, a skin biopsy, which might have contributed significantly to the differential diagnosis, was not performed. Indeed, the importance of performing a skin biopsy should be explicitly highlighted, as it may serve as a valuable diagnostic tool to distinguish between DRESS and azathioprine-induced hypersensitivity syndrome. Although existing data are limited, it has been reported that approximately half of patients experiencing azathioprine-associated hypersensitivity exhibit cutaneous manifestations, with a predominance of neutrophilic dermatoses such as Sweet syndrome [7,20]. On the other hand, skin biopsies from patients with DRESS reveal a variety of nonspecific histologic alterations in the epidermis and dermis, including acanthosis, spongiosis, vacuolization, and the presence of eosinophils and atypical lymphocytes [38,39]. Second, blood PCR testing to detect herpesvirus reactivation, a supportive feature of DRESS syndrome [5,8], was not undertaken. In addition to EBV and CMV reactivation, which has also been observed in patients with azathioprine-associated hypersensitivity reactions [6], human herpesvirus 6 (HHV-6) reactivation occurring approximately three weeks after disease onset is a distinguishing feature of DRESS syndrome and can be assessed either by measuring HHV-6 IgG antibody levels or by detecting viral DNA in blood via PCR. Moreover, HHV-7 may also reactivate simultaneously or sequentially [40,41]. Therefore, performing herpesvirus testing in this case might have provided additional supportive evidence and increased diagnostic confidence in distinguishing DRESS syndrome from azathioprine-associated hypersensitivity reaction.

## 4. Conclusions

Azathioprine-associated hypersensitivity syndrome is a rare, idiosyncratic, dose-independent reaction that typically develops within the first four weeks of therapy. Owing to the presence of systemic symptoms, it may be misdiagnosed as a severe infection or a relapse of the underlying autoimmune disease. Therefore, a high index of clinical suspicion is required, particularly when symptoms occur within weeks of azathioprine initiation. Here, we present the case of a 75-year-old female patient with a highly probable azathioprine-associated hypersensitivity reaction who, in addition to previously reported features, exhibited bihilar and mediastinal lymphadenopathy. This finding may represent a previously unreported manifestation of this syndrome, although an incidental finding unrelated to azathioprine-associated hypersensitivity and attributable to an alternative underlying etiology cannot be excluded.

## Figures and Tables

**Table 1:** Laboratory parameters.

Parameter	Reference Range	Unit	Admission	HD 1	HD 2	HD 3	HD 5	Discharge
Leucocytes	4.0–10.0	×10^9^/L	10.51	12.67	8.59	8.64	10.08	10.42
Neutrophils	33–74	%	83.5	93.6	92.8	86.6	75.3	53.3
Lymphocytes	19–48	%	9.7	2.5	3.7	9.6	17.8	38.5
Absolute eosinophils	<0.5	×10^9^/L	0.02	0.04	0	0	0.01	0.02
Hemoglobin	120–160	g/L	140	135	118	123	121	133
Platelets	150–450	×10^9^/L	238	232	208	261	261	272
INR	<1.2		1.3					1.0
Sodium	136–146	mmol/L	133	130	130	134	135	137
Potassium	3.5–5.1	mmol/L	3.7	3.6	3.5	4.0	3.9	3.8
Creatinine	45–84	µmol/L	86	95	87	72	71	79
eGFR	>90	mL/min/1.73 m^2^	57	51	57	71	72	63
ASAT	<31	U/L	59	80	49	33	29	22
ALAT	<31	U/L	63	60	46	40	36	26
ALP	53–141	U/L	174	284	185	182	164	109
Gamma-GT	<38	U/L	406	622	462			237
LDH	<247	U/L	375	368				
Lipase	<60	U/L	405	262	88			161
Total bilirubin	1.7–21.0	µmol/L	38.3	50.3	37.9			15.8
Albumin	35–52	g/L	34		26	28	27	35
CRP	<5	mg/L	122	197	201	80	27	3.9
Procalcitonin	<0.5	µg/L	1.0					

ALAT: Alanine aminotransferase; ALP: Alkaline phosphatase; ASAT: Aspartate aminotransferase; CRP: C-reactive protein; eGFR: Estimated glomerular filtration rate; Gamma-GT: Gamma-glutamyl transferase; HD: Hospital day; INR: International normalized ratio; LDH: Lactate dehydrogenase.

**Table 2:** Clinical parameters, diagnostic considerations, and differential diagnosis.

Category	Findings
Patient characteristics	75-year-old female; autoimmune hepatitis; type II diabetes mellitus
Drug exposure	Azathioprine and antibiotic therapy initiated approximately 3 weeks prior to symptom onset
Clinical symptoms	Fever, emesis, maculopapular rash (lower legs and palms), arthralgia (knees, heels), lumbar back pain
Gastrointestinal/hepatic/renal findings	Elevated transaminases, cholestatic parameters, elevated lipase, elevated creatinine
Systemic/inflammatory markers	Elevated CRP; elevated PCT; near normal leukocyte count; no eosinophilia
Imaging findings	CT: hilar and mediastinal lymphadenopathy; no infectious focus or biliary obstruction
Treatment response	Rapid improvement after azathioprine discontinuation and corticosteroids
Primary diagnosis	Azathioprine-induced hypersensitivity reaction (Type B drug hypersensitivity reaction)
Differential diagnosis	DRESS syndrome (RegiSCAR score 5; atypical due to absence of eosinophilia), sarcoidosis (excluded), systemic infection (excluded)

CRP: C-reactive protein; CT: Computed tomography; DRESS: Drug reaction with eosinophilia and systemic symptoms; PCT: procalcitonin.

## Data Availability

No new data were created or analyzed in this study. Data sharing is not applicable to this article.
